# Patient perspectives

**DOI:** 10.1016/j.lanwpc.2025.101558

**Published:** 2025-06-29

**Authors:** Carmel R. Crook, Sandra C. Thompson

**Affiliations:** a The Sax Institute, 30C Wentworth Street, Glebe, New South Wales 2037, Australia; b School of Medicine and Public Health, The University of Newcastle, University Drive, Callaghan, New South Wales 2380, Australia; c Western Australian Centre for Rural Health, University of Western Australia, 167 Fitzgerald Street, Geraldton, Western Australia 6530, Australia

We have spent many hours with First Nation people, hearing stories about their journey with cancer. So many stories are profound, moving and provide testimony to courage, frustration, support from family, while noting both excellent care and failures in achieving seamless integrated care. As researchers we have disseminated those stories and reflected upon them to inform better approaches to cancer prevention and care. The insights provide a strong rationale for guidelines that incorporate a consumer perspective into research and patients’ care.

Many experiences of cancer across the spectrum of prevention, diagnosis and care are shared by individuals, regardless of whether they are First Nations or have other cultural backgrounds. We all share a common humanity and vulnerability, especially at times of illness. Through our shared experience and understanding of differences, we can learn to better appreciate others’ problems and deepen understanding and empathy towards the many challenges people experience. In addition, given their ongoing poor health outcomes, there are extra considerations for First Nations, particularly issues related to cultural security, general health, health literacy, caring responsibilities for (extended) family and racism.

Despite significant advances in cancer knowledge and treatments, the gap between cancer mortality rates of First Nations and non-First Nations Australians is widening, with age standardised mortality rates from 2006 to 2019 increasing by 14% for First Nations and decreasing by 13% for other Australians over this period.^1^ This is unacceptable and demands change.

The Australian population is intentionally exposed to multiple messages through campaigns such as *Find Cancer Early*, some directed specifically at First Nation peoples, recommending age-appropriate screening and for individuals to not ignore symptoms. The underlying message is that acting upon abnormalities or symptoms early will result in better outcomes once a cancer is diagnosed. That is, if found early, cancer can more likely be treated and even cured. This is a clear message, easy to understand and believable.

So how distressing if, following detection of an abnormality, a protracted period of waiting ensues to get expert specialist input and a definitive diagnosis—and then for the recommended treatment to begin. The literature is replete with patients’ stories of challenges and delays they face from detecting an abnormality to entering treatment,^2^ an issue particularly likely for First Nations patients.

Two vignettes of patients facing challenges with diagnosis and referral to get a more definitive diagnosis, illustrate the uncertainty of what an abnormality means and the angst and worry of time delays while waiting for clarification and for treatment to begin.

The first vignette, provided by an educated metropolitan based First Nations person with private health insurance, shows the cancer journey into treatment is daunting regardless of knowledge and ability to access the private health system. She describes two cancer diagnoses 10 years apart. This vignette highlights how both trust and systemic inefficiencies can shape a patient’s experience. While both journeys involved dedicated and capable healthcare professionals, the processes and structures surrounding them varied significantly.

## Vignette 1

**First cancer journey: trust, connection, and a coordinated pathway**

The first time I was diagnosed with cancer, I was unfamiliar with the territory, navigating a complex and frightening world largely for the first time. Yet, despite the fear, my experience was anchored in trust. At the time, I had an established relationship with a specialist for another chronic condition and this relationship prompted early action. I had reported vague but concerning symptoms, and rather than dismiss them, the specialist organised a PET scan. The results revealed an urgent need to commence treatment.

While the diagnosis was distressing, it was delivered with care and sensitivity. The specialist ensured my partner was present during that first crucial consultation, explained the findings clearly in plain language, outlining both the seriousness of the diagnosis and the steps ahead. His communication was patient-centred—balanced, compassionate, and free of unnecessary medical jargon. I was presented with options, and a treatment plan was mapped collaboratively.

This relationship enabled both my agency and sense of security. I trusted this clinician and the process he recommended. He listened, galvanised the team around him, coordinated hospital processes with efficiency and effectiveness, and prepared me and my family for the difficult journey ahead. Decisions were made with me, not for me. The experience, though emotionally and physically challenging, felt coordinated and purposeful.

**Second cancer journey: fragmentation, delays, and disempowerment**

A decade later, I had a second, unrelated cancer diagnosis. I entered this second journey with more health literacy, a clearer understanding of cancer pathways, and a stronger sense of agency. Yet, paradoxically, this journey left me feeling marginalised and excluded from critical aspects of my own care.

Although cancer was suspected early, the investigative pathway was convoluted. Attention was initially directed towards other possible causes—nerve issues, head injury, and other differential diagnoses. Several distressing months elapsed between the initial identification of a sizeable tumour and the commencement of treatment.

The initial delay was compounded by the structural challenges of coordinating multiple teams across different specialties, seeming to operate in silos, often appearing to be at cross-purposes rather than working as a unified team. Decisions were made about my health without my involvement or, at times, even my awareness. In key moments, I found myself feeling like a passive observer, watching the system move sluggishly around me.

A devastating consequence of these delays was missing out on clinical trial eligibility—an opportunity that may have extended my life or improved my quality of life. I was caught in a bureaucratic bottleneck, with logistical challenges and inter-team miscommunication eroding my trust in the system I had no choice but to rely on.

Emotionally, my second journey felt less caring and more transactional. Sometimes, sensitive information was delivered by locum doctors with whom I had no established relationship. On several occasions, distressing news was conveyed without any effort to arrange for a support person to be present. Medical language was sometimes used insensitively or without context — for example, references to “palliative care” were made with little explanation, creating fear and confusion around my prognosis. The delays and lack of continuity in the people delivering critical information, left me feeling excluded from decisions that directly affect my survival and quality of life, impacting my overall wellbeing and that of my family.

**People vs. process: a patient’s perspective**

Across both experiences, the dedication of individual clinicians remained largely consistent. Most of the healthcare professionals I encountered were conscientious and compassionate. However, the broader processes that shaped each journey diverged sharply.

In the first journey, trust was underpinned by strong, direct relationships and a sense of continuity. Communication was inclusive and deliberate, supporting a shared understanding of what was happening and why. I felt respected and engaged, and despite the gravity of the diagnosis, I felt there was a collaborative purpose to achieve the best outcome possible for me. In the second journey, despite well-intentioned clinicians, the system was fragmented, with no clear anchor of an informed person guiding me. The cancer care coordinator was overstretched. The timing of my entry into the system during the end-of-year period with skeleton staffing exacerbated the delays. I was acutely aware of the severity of my diagnosis, yet my sense of urgency did not appear to be met with a clear process to ensure continuity; decisions were delayed, communications fractured, and critical windows of opportunity were lost.

In reflecting on these two journeys, it is apparent is that compassionate people will not be enough for improving cancer outcomes; systems that work smoothly and predictably are essential. In the absence of timely processes and coordinated care, even the most empathetic individuals cannot compensate for the emotional toll of systemic inefficiencies.

*Ultimately, my story reflects a broader truth in healthcare: patients don’t just experience disease—they experience the system. And the system’s ability to deliver timely, coordinated, and person-centred care makes all the difference*.

The second vignette is abbreviated from a longer description provided by the daughter of an elderly woman who initially lived on Country, hundreds of kilometres away. She described the experience of her mother and her path to diagnosis and treatment, a patient journey over many years and through which many inadequacies in the system of care were evident.

## Vignette 2

Mum lived in a remote community serviced by a nurse practitioner. A doctor visited periodically but there was no continuity with the provider. It started with cough, attributed to her blood pressure medication being too strong. It was the local pharmacist who noticed the cough was persisting and told her she must see the doctor. She had a wheeze that was progressively getting worse over a couple of years, she was getting really bad. Pretty much struggling to breathe and nothing different was happening through the local health providers. So, I insisted she come to me in the capital city as her prescribed asthma puffer wasn’t working.

She ended up in hospital with pneumonia, was almost overdosed because the health team did not listen to the medications she was taking. “*I realised the health system is shot to pieces so much, I was there morning, noon and night*”.

After 2–3 weeks in hospital, she had recovered from the pneumonia and was discharged. A few weeks later she has a clinic appointment, and they kept asking us if she had had tuberculosis or had been to an Asian country in the last couple of years. The abnormality was still there, and they said “it's probably caused from the buildup of mucus and things in her lung. So, we'll see how it clears up over a couple of months”. With the next check-up weeks later, the doctor was happy … then said, “Look, there is still a mass there, we will get you back in to have more scans”. We waited three months for them to send it.

So, I talked to my own doctor; he sent her straight up for the scan–then they said you need to get to see your doctor immediately. The scans showed a tumour in her lung, this one had grown so large it was blocking her airway–it was almost grown fully across where your lungs separate into the right and left lung cavity. They tried three times with a snare to remove the lesion, but the third time collapsed her lung.

She almost died. Multiple simultaneous Code Blues were happening. I called the nurse. My mum never complains, she's as tough as, but she was going like this and rocking in the bed. And I knew that something serious was wrong. She was not being monitored, and she was not getting attention. I was watching the monitors; everything was going pear shaped. So, I ran to find that nurse, she’d got distracted with another Code Blue. And I actually had a big growl at her. Mum was going downhill very rapidly. Only my demanding resulted in getting her attention–that led to immediate emergency surgery. She was in surgery for 5 h. She pulled through that. After she needed further surgery–her whole right lung removed. She was in ICU for a long time … it was touch and go. In the ward for month, it was a long recovery, she's never been the same. It had deconditioned her so badly. I left work to look after her. I was by her side, morning, noon and night, I was too frightened to leave her. Because every time I would go home something would happen.

I’m critical of the many failures, starting with failures in primary health care, lack of continuity and the lack of chest x-ray in the remote primary care setting. We requested all our mum’s previous medical history. And, sadly to say, after all the years Mum lived in <the town>, there was only two pages with some scribbled notes. There wasn't even a diagnosis of asthma, just a prescription for the puffer at that stage, no diagnosis, and no recommendation to do further testing. Multiple times there was delay in diagnosis and treatment–as well as mum nearly dying because she was unmonitored at a critical time. The Aboriginal liaison staff in the hospital were hard to get hold of. The experience with the delay in diagnosis and treatment failures meant mum required multiple operations, became frail and her cognitive function declined—the whole treatment process, all the anaesthetics, she's not understanding as well as she used to.

*[We needed] a team who were well equipped, well informed and absolutely understood the essence of Aboriginal culture. And just basic human respect, and knowledge of the systems, that would make have made all the difference in the world to me at times. I felt so despairing ….”*

Stories of delayed diagnosis have too often been attributed to First Nations people’s late presentation, considered to reflect low health literacy because the individuals didn’t recognize abnormalities that could be associated with cancer. The temptation to blame the individual takes the heat off scrutinising an inequitable system for whether it promotes or undermines people’s capacity to make optimum decisions about care during their cancer journey. Yet, there is good evidence captured from multiple First Nations testimonies of many failures of diagnosis when people seek care.^2^ And despite the care and support from individual practitioners often being very good, there is little appreciation *by the system* of the fear and concerns that patients’ experience at the time of help-seeking. So, treatment delays can feel intolerable. It is time to stop blaming the victim and look critically at how systems function and connect to deliver the care that people need. At the heart of these stories is not the issue of health literacy but *a system of care not aligned to the needs of the patients it needs to serve*.

We must reflect on where problems lie, given delays in referral for an identified abnormality to definitive diagnosis and treatment, and the consequences for the patient. In Australia’s two-tier health system, public and private, there is under-investment in public hospitals.^3^ In the public system, patients are effectively powerless in the face of triaging and waiting lists which lead to delays at a critical time. While access to specialists and many treatments is available more quickly in the private system for those who can afford it, this is not a panacea for well-integrated timely care, even for the 20% of First Nations patients who have private health insurance. Inequality in access to timely quality care is real.

Time is of the essence and delays result in huge stress, particularly when we are vulnerable and aware of our own mortality. Especially for those whose needs are complex, better joined up care is needed; care that is responsive to their needs as a whole person and not a body parts or single tumour approach. Care should not be a series of poorly connected episodic appointments. While “patient centred” is often spoken about, our vignettes highlight the lack of seamless integrated timely care across the health care system.

There is so much commonality in the issues raised by First Nations people over time and across different jurisdictions and settings. The legitimacy of the consumer voice is now recognised in multiple policy documents and there is strength and integrity in insights gained from lived experience that can inform translation of findings into improvements in policy and practice.^4^ Cancer Australia has taken important steps towards achieving this.^5^ We must do better. We look forward to a responsive health system that redesigns itself around patients’ needs, including respectful inclusion and attention to all the issues in treatment and care raised by Australian First Nations people.

## Contributors

Conceptualisation–CC, ST; Writing—original draft CC, ST; Writing—review & editing CC, ST.

## Declaration of interests

The authors have no conflicts of interest to declare.
